# Role of leaders’ positive mindset in mitigating the effects of crises on organizations: The case of Canadian organizations

**DOI:** 10.1371/journal.pone.0319931

**Published:** 2025-03-21

**Authors:** Mohammed Laid Ouakouak, Noufou Ouedraogo, Gertrude I. Hewapathirana, Michel Zaitouni

**Affiliations:** 1 GUST Center for Sustainable Development (CSD), Gulf University for Science and Technology, Hawally, Kuwait; 2 Department of Management & Organizations, School of Business, Grant MacEwan University, Edmonton, Canada; 3 Business and Management Department, Knox College, Galesburg, Illinois, United States of America; Universiti Pertahanan Nasional Malaysia, MALAYSIA

## Abstract

Maintaining a positive mindset is important for leadership effectiveness and may even be more important in times of a crisis because such a mindset contributes to organizational survival and resilience. In this study, we examined whether leaders’ positive mindset helped to mitigate the harmful effects of the COVID-19 crisis on organizations and if so, how. To address this issue, we conducted an empirical study through LinkedIn with 165 participants working in various organizations in Canada. The results show that leaders’ positive mindset positively impacts leaders’ innovative behavior. However, leaders’ innovative behavior did not attenuate the negative effects of the COVID-19 crisis on organizations, except when we introduce the use of information and communication technology (ICT) and the provision of psychological support to employees as moderators. The implications of these findings for both theory and practice, as well as some future research directions are discussed.

## 1. Introduction

Scholars’ and practitioners’ interest in the concept of organizational survival has grown in recent years in response to increasing environmental uncertainties and unexpected crises. Indeed, organizations today face unprecedented challenges due to crises such as the COVID-19 pandemic, which has disrupted global markets and organizational structures. Therefore, business leaders and managers are looking for ways to survive during crises and limit their damaging effects [1,2]. The COVID-19 crisis has caused extreme disruption and uncertainty [3] and created serious challenges that have profoundly affected the operations and performance of organizations [4–6]. One observation related to organizational outcomes following the COVID-19 pandemic has captured the attention of scholars: some organizations were able to navigate the crisis more successfully than others. This raised a natural question: What factors helped organizations to survive and to mitigate the negative effects of the COVID-19 crisis? The current research aimed to address this question.

One of the most important ingredients to successfully manage a crisis facing an organization is the positive mindset of its leaders [7,8], so our research considers this aspect as the starting point. Leadership is one of the most critical internal factors of an organization’s survival during a crisis [8]. Indeed, in a crisis, leaders are expected to exhibit a unique set of skills and abilities that differ from those they apply during routine operations [9]. For example, scholars have reported that organizations’ survival and success in times of crisis are associated with leaders’ abilities and characteristics, such as risk-taking [10], self-confidence [11], and optimism [12].

Although prior research has examined the role of certain leadership characteristics in rebuilding organizations in times of crisis, scholars have called for more studies to better understand the way leaders impact organizational survival and success during a crisis [13,14]. Leaders are role models and as such, followers look up to them for reassurance, direction and guidance, and optimism and positivity when their organization faces a crisis. We believe that leaders who exhibit confidence, composure and courage will likely inspire the same in their followers. As such, a positive mindset may be one of the most critical leadership qualities that affect the ability of an organization to successfully navigate a crisis.

A leader’s positive mindset is a state of mind during which leaders are filled with confidence, optimism and positivity, that are reflected in their behaviors and actions. Indeed, the literature highlights the critical role of positive mindset in crisis management [8]. For example, leaders with a positive mindset are more likely to absorb the negative effects of a crisis and transform these effects into opportunities for their organizations [15]. By showing confidence and optimism during a crisis, leaders contribute to effectively managing it [16].

We therefore endeavor to uncover how leaders’ positive mindset helped organizations mitigate the effects of a crisis, specifically the effect of the COVID-19 pandemic in Canadian organizations.

Innovation is another crucial factor that helps organizations cope with the adverse effects of a crisis [17]. Innovation is another vitally important strategic tool used by companies to respond to the challenges and risks caused by crises [18].

A crisis like the COVID-19 pandemic presents new and unique challenges to organizations which cannot be effectively addressed with routine solutions and therefore require innovative solutions. The research findings suggest highly innovative companies are more likely to survive and even grow in times of crisis compared to less innovative organizations [19–21]. As situational leadership theory suggests, successful leaders tend to counteract the negative effects of a crisis by adopting innovative approaches and reshaping their behavior accordingly [7,22]. Drawing on this theory and on theoretical arguments presented in the literature related to leaders’ positive mindset, innovation, and crisis management, we aim to empirically test the impact of leaders’ positive mindset on their innovative behavior, and then, the impact of that innovative behavior on the effects of the COVID-19 crisis. We contend that maintaining a positive mindset during a crisis provides leaders with the degree of determination, confidence and optimism they need to adopt innovative behaviors, which, in turn, reduces the negative effects that the crisis has on their organization [7]. Ultimately, our purpose is to contribute to theory on crisis management and to make practical recommendations to leaders on how to successfully navigate crises to ensure survival and recovery of their organizations.

In the next section, we first present the theoretical underpinnings of our paper, and we justify our hypotheses.

## 2. Literature review and hypotheses

The extent literature shows many benefits associated with a leader’s positive mindset. Using a variety of theoretical lenses, Avery et al. [23] have emphasized the value of a positive mindset in leaders and have explained how such a mindset sustains an organization in the contexts of a crisis. These theoretical lenses provide frameworks for understanding the benefits of a leader’s positive mindset on organizational outcomes, employee well-being, and overall effectiveness. For example, positive leadership theory indicates that a positive mindset is fundamental to leaders’ ability to both adjust their behaviors in crisis situations and foster trust and hope among employees [24]. Leaders who possess positive mindsets maintain positive emotions, engagement, and relationships, and foster hope and optimism to inspire teams to enhance the performance and well-being of others [25]. Leaders who display a growth mindset can be convinced that crisis not only brings challenges but also generates opportunities to initiate innovative solutions [26]. In doing so, those leaders recognize employees’ strengths to boost engagement and creativity by solving problems under pressure [27], which can foster collective efficacy and inspire employees to innovate and enhance performance [28].

Positive leadership theory indicates that a positive mindset is fundamental to a leader's ability to both adjust their behaviors in crises and foster trust, hope, and confidence among employees [24]. A leader's positive mindset has been viewed as the most valuable intangible resource because it can significantly influence the way that a leader navigates and leads to upholding organizational survival during a crisis [29].

A leader’s positive mindset is one of the most valuable intangible resources because it can significantly influence the way that leader navigates through a crisis to ensure organizational survival [29]. Leaders who possess a positive mindset envision success, so they make consistent efforts to amplify a positive work environment, encourage an optimistic work culture, and enhance innovation, while adapting their behavior to specific situations, and serving as catalysts in crisis contexts [24]. A leader’s positive mindset therefore instills confidence and stability within employees and stakeholders and enhances adaptability and innovation in the organization [30]. More importantly, leaders can use their positive mindset to transform crises into favourable organizational renewal opportunities [31]. A leader’s positive mindset instills confidence and stability within employees and stakeholders and enhances adaptability and innovation in the organization [30]. More importantly, leaders can use their positive mindset to transform crises into favorable organizational renewal opportunities [31].

Situational leadership theory suggests that leaders demonstrate flexibility in their leadership styles, by directing, coaching, and supporting employees by empowering teams during the crisis process [32]. Furthermore, technological adaptation models and diffusion of innovation theories provide a framework for understanding how leaders adopt and leverage technology during a crisis [33]. These theories explain how leaders assess the perceived usefulness of various technologies and processes and adopt them to accelerate innovations, thus fostering a culture of innovation during a crisis to mitigate the harmful effects to facilitate future growth [33,34].

Similarly, positive psychology enables leaders to foster psychological safety while employees feel confident in suggesting innovative ideas. In such an event, situational leadership abilities further complement employees feeling empowered and motivated to contribute [25]. Along the same vein, situational leadership complements leaders’ continuously shifting strategies throughout the crisis process. Based on these theoretical perspectives, we argue that by integrating positive psychology, situational leadership, and technological adaptation theories, leaders enhance their capacity to adapt to crises and foster innovation. While positive psychology enables emotional and psychological resilience, technological adaptation theories enable leaders to integrate innovative technological tools to mitigate the harmful effects of the crisis [33,34].

### 2.1. Leaders’ positive mindset and leaders’ innovative behavior

The link between a leader’s positive mindset and innovation is supported by the dynamic capabilities theory, which emphasizes the ability of organizations to integrate, build, and reconfigure internal and external competencies to address rapidly changing environments [35]. Leaders with a positive mindset leverage these dynamic capabilities by fostering innovation—a critical strategy for adapting to and overcoming crisis-induced disruptions. Through innovative behaviors, leaders drive the creation of novel solutions that enhance organizational resilience and performance [18,19].

Research shows that leaders’ positive mindset, and leaders’ innovative behavior are indeed some of the factors that helped organizations operate and constantly adapt during the COVID-19 pandemic [36].

Leaders with a positive mindset foster innovation and mitigate the effects of various problems that arise in uncertain times through creative and highly innovative behaviors [37]. Leaders who demonstrate a positive mindset quickly assemble resources needed for innovation while envisioning new practices to engage their employees in the recovery process [38]. Positive-minded leaders are more likely to develop creativity and innovation capabilities and foster a creative climate among their followers [37]. Such leaders also display a growth mindset as they continuously exhibit new ways to solve problems, thereby positively influencing organizational innovation [39].

Leaders who possess a positive mindset also have been observed engaging into product/market innovations and taking the lead to salvage their organizations during crisis situations [26,40]. These leaders are willing to integrate risky innovations and to proactively experiment with new technology and opportunities to facilitate the growth of their organizations.

The literature shows that leaders who possess a positive mindset are more likely to foster creativity, promote a climate conducive to success for organizational members, and encourage those members to innovate and implement solutions to mitigate the harmful effects of crises [41]. Building on the literature reviewed thus far, we hypothesized the following in the context of COVID-19.

H1. Leaders’ positive mindset has a positive effect on leaders’ innovative behavior.

### 2.2. Mitigating the effects of covid-19 on organizations through leaders’ innovative behavior

Leaders who exhibit innovative behaviors adapt quickly to unexpected situations, and excel in crisis management and problem-solving [42]. They foster innovation and facilitate their organization’s adaptation and responsiveness to reduce the negative impact of a crisis [43].

In crisis situations, leaders’ innovative behavior therefore plays a pivotal role in mitigating negative effects while positively responding to unexpected circumstances [44].

During the COVID-19 pandemic specifically, leaders had to reimagine all aspects of their organization’s functions and business processes and put in place creative measures to provide practical and implementable solutions to address the challenges created by the crisis [45]. These innovations enabled leaders to adapt to uncertainties created by the pandemic [46]. Technological adaptation and situational leadership theories suggest that leaders can swiftly enhance their capacity to adapt to crises and foster innovation by integrating innovative technology [33,34]. Pioneering positive-minded leaders quickly restructure their distribution services to minimize supply chain shortages and to make distribution systems more efficient to address urgent customer needs [21]. Innovative leaders quickly responded by allocating resources, implementing new ideas and using information related to the pandemic to reduce its effects [47]. Therefore, we hypothesized that.

H2. Leaders’ innovative behavior is negatively associated with the negative effects of COVID-19 on their organizations.

### 2.3. ICT as a moderator in the relationship between leaders’ innovative behavior and the effects of COVID-19 on organizations

Information and communication technology (ICT) is one of the most important tools that organizations use to operate in complex and uncertain business environments [48]. ICT serves as a critical enabler for maintaining trust and collaboration during crises, allowing leaders to disseminate information effectively and coordinate innovative initiatives [49,50].

Leaders who embrace technological adaptation can also integrate digital tools to innovate during crises [33,34]. For example, during the COVID-19 pandemic, leaders leveraged virtual collaboration tools (e.g., Zoom, Microsoft Teams) to maintain operations remotely and develop new business models. Their positive mindset ensured teams stayed motivated during the transition, while situational leadership provided the flexibility to manage varying levels of technological proficiency within the team.

During the crisis, ICT was crucial in facilitating the remote collaborations and interactions between leaders and employees and for nurturing resilience among leaders and other stakeholders [49]. Guzzo and colleagues [50], using the leader–member exchange (LMX) theory perspective, discussed leadership crisis communications during the COVID-19 pandemic and emphasized that ICT, which was used as an effective means for communicating with employees during the crisis, was essential to developing favorable attitudes. Leaders tended to use ICT because they believed that constant and high-quality communication enhanced interpersonal relationships and trust with employees at all levels [15,36].

The pandemic has also opened many opportunities for new ideas, goods and services, including digitization, and the latter proved to be a highly effective tool facilitating the flow of ideas and information [47]. In fact, leaders who demonstrated a positive mindset also tended to quickly embrace digitization [47].

Likewise, sophisticated digital technology, such as big data analytics and artificial intelligence, helped leaders overcome the negative effects of the pandemic [51,52]. Based on the research findings reported above, we hypothesize the following:

H3. The use of ICT moderates the relationship between leaders’ innovative behavior and Covid-19 effect.

### 2.4. Psychological support to employees as a moderator in the relationship between leaders’ innovative behavior and Covid-19 effect

Providing psychological support to employees helps with organizational resilience during crises [53]. Psychological support refers to managerial efforts to provide employees with emotional support for work engagement and opportunities to improve their task performance, as well as to provide them with resources, information, learning, growth opportunities, and motivation [54].

Psychological support to employees is a mechanism to enhance their agency and resilience during crises [55]. Leaders who prioritize employees’ psychological well-being foster a supportive environment that enables teams to engage in innovative behaviors and contribute to organizational recovery and growth. Psychological support enhances employees’ ability to manage stress, maintain productivity, and align with innovative strategies during turbulent times [56,57].

The provision of psychological support and resources to mitigate the negative impact of a crisis on employees is paramount to maintaining their well-being and mental health during such stressful times [58]. Under crisis conditions, leaders’ psychological support plays a vital role in maintaining employees’ morale, commitment to their work and confidence in their leaders [53,55]. Scholars advocate that leaders should pay attention to their employees to better understand their emotions, attitudes and concerns [59] and then build the employees’ trust and confidence to best cope with crises [16].

Leaders’ innovative behavior, especially when coupled with the provision of adequate psychological support to employees, is therefore positively associated with organizational survival [53]. A leader’s ability to persuade employees and to infuse positivity and excitement among them is crucial to managing crises [55], helping the leader to implement innovative initiatives more easily by garnering support at all levels. Therefore, we hypothesize the following:

H4. Psychological support to employees moderates the relationship between leaders’ innovative behavior and the effects of the Covid-19.

**Fig 1:**
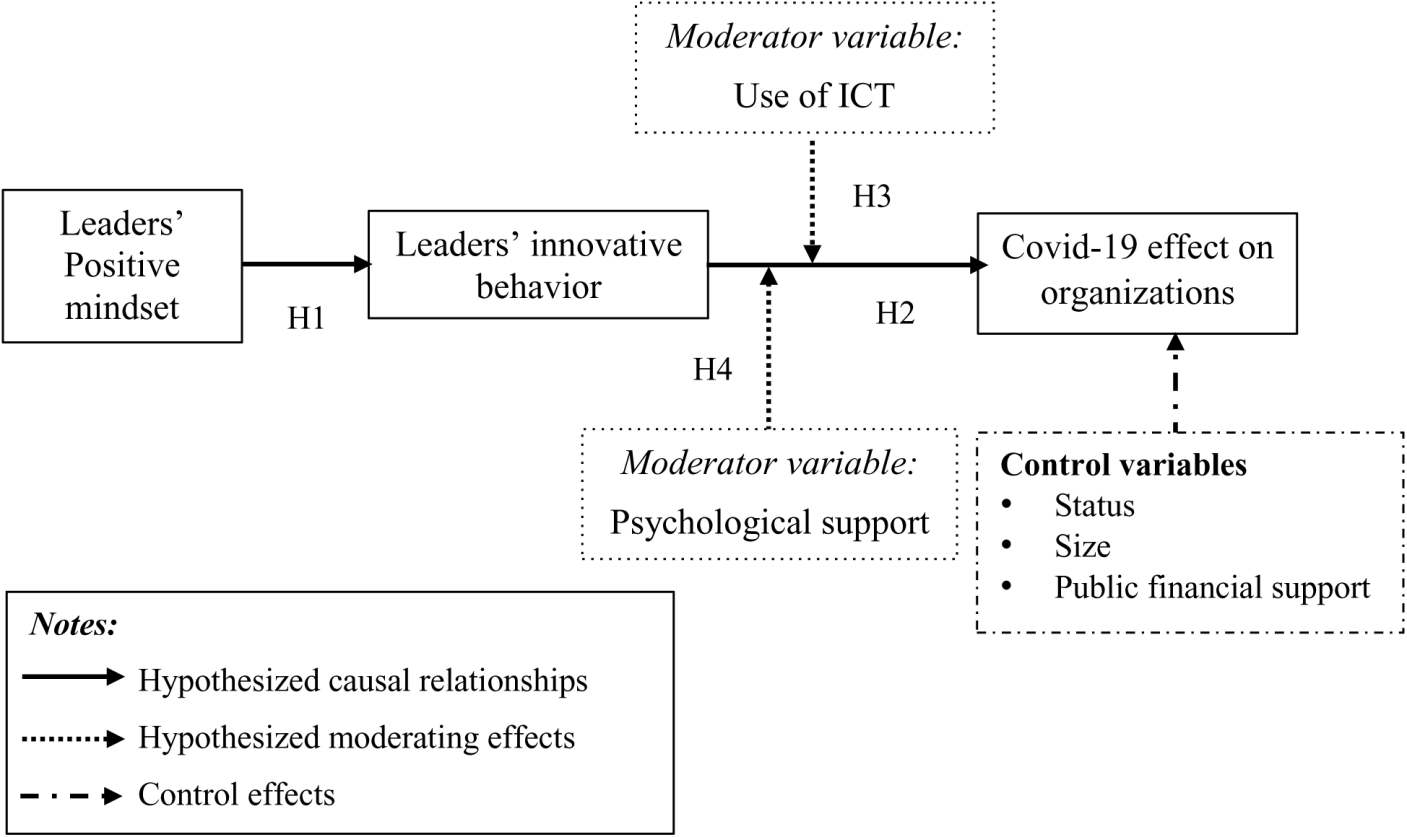
Research model.

Fig 1 details the hypothetical relationships.

## 3. Research methodology

### 3.1. Data collection

This study is part of a larger research project investigating how organizations adapt and build resilience to mitigate the negative effects of a crisis, such as the COVID-19 pandemic crisis. To collect data for this study, we created a survey questionnaire, which we uploaded onto www.surveymonkey.com, and we recruited participants to complete the survey through one of the authors’ LinkedIn network. These participants consisted of professionals who worked in various companies in Canada. As a professional networking website, LinkedIn provides such information as a member’s job title, affiliations, education, qualifications and professional experience. As such, it serves as a good source of data for purposive sampling [60] because prospective participants can be targeted based on their profiles and the likelihood that they have the necessary experience and information to answer the research questions.

We sent personalized messages that included the survey link to potential participants to invite them to take part in the study. The consent form served as the first page of the survey, and included various details, such as contact information for the researchers and the research ethics board, the research procedure, potential risks and benefits of the study, confidentiality and anonymity assurances, and the respondents’ right to withdraw from the study or to skip any questions they did not feel comfortable answering. Of the pool of approximately 1,600 potential participants contacted, 165 participants submitted complete responses that supplied the data for our analysis, reflecting a response rate of 10.31%. The descriptive statistics of this sample are presented in Table 1.

**Table 1:** Data description.

Characteristics	Frequency	Percentage
Organization size:		
0: self-employed	10	6.1
1 -9 employees	18	10.9
10-49 employees	36	21.8
50-249 employees	22	13.3
250-499 employees	12	7.3
500 or more employees	67	40.6
Firm status:		
For profit business	107	64.8
Nonprofit organization	58	35.2
Industry:		
Primary sector (*Mining and other natural resources, fishing, agriculture, etc.*)	12	7.3
Secondary sector (*Manufacturing, construction, etc*.)	32	19.4
Tertiary sector (*Transportation, banking, education, communication, etc.*)	121	73.3

### 3.2. Measures

We developed all measures of the constructs based on the literature, interviews with practitioners and consultations with academics. Respondents were asked to indicate their level of agreement with each statement on a five-point Likert scale (from 1 =  strongly disagree to 5 =  strongly agree).

*Leader’s positive mindset* was measured using a scale that we developed for the study, with a sample item as follows: “During the pandemic, leaders of my organization had a positive mindset, even when facing difficulties”. *Leaders’ innovative behavior* was assessed with a five-item scale, with a sample item as follows: “During the pandemic, the leaders of my organization developed innovative solutions to our organization’s problems”. *Covid-19 effect on organizations* was assessed with a five-item scale. The participants were asked whether the pandemic had negatively affected their organization’s internal operations, productivity, market share, financial performance, and growth.

*Use of information and communication technology (ICT)* was measured on a four-item scale, with a sample item as follows: “during the Covid-19 pandemic, my organization effectively used the Internet or other remote work technologies”. *Psychological support* was measured using a five-item scale, with a sample item as follows: “during the pandemic my organization provided psychological support to employees”. All the constructs’ measures are provided in Appendix A.

We controlled for the following variables that might covary with the dependent variable “*COVID-19 effect*”: organizational size (0 =  small company with fewer than 250 employees; 1 =  large company with more than 250 employees), status (0 =  for-profit business, 1 = nonprofit organization), and public financial support (two groups were created using the median of the computed 5-item variable: 1 for low public support and 2 for high public support). We anticipate that these control variables may interact with Covid-19 effect on organizations.

First, public financial support was controlled for, as organizations receiving external financial aid may demonstrate different levels of resilience compared to those without such support. Second, organizational size was included as a control variable because larger organizations often have more resources, structured processes, and established crisis management strategies, which may enable them to better mitigate the effects of a crisis compared to smaller entities. Finally, organizational status (profit versus nonprofit) was included as a control variable due to differences in operational priorities and funding mechanisms, which could influence their responses and recovery processes during the Covid-19 crisis. By controlling for these variables, the study ensured that the observed effects were more accurately attributed to the leaders’ positive mindset and leaders’ innovative behaviors rather than extraneous factors.

## 4. Data analysis and results

### 4.1. Descriptive statistics and correlations

Table 2 presents the means, standard deviations and correlations for all variables. Consistent with our arguments, leaders’ positive mindset is positively associated with leaders’ innovative behavior (*r* =  .655, *p* =  .01) and negatively associated with the pandemic effects (*r* =  − .231, *p* =  .01), while leaders’ innovative behavior is negatively associated with the pandemic effects (*r* =  − .169, *p* =  .05). Regarding the control variable effects, we found that none had a significant impact on the Covid-19 effect. These results provide preliminary evidence to support our research hypotheses.

**Table 2:** Means, standard deviations and correlations among research variables.

N	Constructs	Mean	Std. Dev.	1	2	3	4	5	6
1	Financial support	1.480	.501	1					
2	Status	1.351	.479	.057	1				
3	Organizational size	1.478	.501	.053	.031	1			
4	Leaders’ positive mindset	4.132	.683	.166^*^	−.031	−.021	1		
5	Leaders’ innovative behavior	4.137	.901	.132	−.019	−.098	.655^**^	1	
6	Covid-19 effect on organizations	2.824	1.120	.095	.129	−.152	−.231^**^	−.169^*^	1

*Correlation is significant at the 0.05 level (2-tailed).

**Correlation is significant at the 0.01 level (2-tailed).

### 4.2. Reliability and validity assessment for the scales

Before testing the research hypotheses, we ran a confirmatory factor analysis (CFA) using AMOS 25.0 to assess the reliability and validity of the measurement scales. The measurement model consisted of leaders’ positive mindset, leaders’ innovative behavior and Covid-19 effect. The CFA offered the following indices, indicating a good model fit [61]: chi-square divided by the degree of freedom (χ2/*df*) =  1.893; comparative fit index (CFI) =  0.932; Tucker-Lewis index (TLI) =  0.922; incremental fit index (IFI) =  0.933; and root mean square error of approximation (RMSEA) =  0.074. Table 3 reports factor loadings with statistical significance (*p* <  .001), illustrating that all factor loadings were higher than the recommended value of 0.60 [62], except for the item “IU1.” We decided to keep this item in the analysis due to significant loading.

**Table 3:** Results of CFA analysis.

Constructs/indicators	Standardized factor loadings
**Leaders’ positive mindset**	
LEAD-MIN1	726
LEAD-MIN2	.800
LEAD-MIN3	.782
LEAD-MIN4	.783
**Leaders’ innovative Behavior**	
INN-BEH1	.868
INN-BEH2	.866
INN-BEH3	.863
INN-BEH4	.911
INN-BEH5	.924
**Covid-19 effect on organizations**	
PAN-EFF1	.685
PAN-EFF2	.639
PAN-EFF3	.771
PAN-EFF4	.891
PAN-EFF5	.867
**Use of ICT**	
ICT-USE1	.577
ICT-USE2	.706
ICT-USE3	.797
ICT-USE4	.871
**Psychological Support**	
PSY-SUP1	.849
PSY-SUP2	.926
PSY-SUP3	.845
PSY-SUP4	.848
PSY-SUP5	.894

We further assessed the reliability and validity (i.e., convergent validity) of the scales by calculating the Cronbach’s alpha, construct reliability (CR), and average variance extracted (AVE). As displayed in Table 4, all values exceed the recommended levels of 0.70 for Cronbach’s alpha and CR and of 0.50 for AVE, confirming adequate reliability and convergent validity for all constructs in this study [61].

**Table 4:** Reliability and convergent validity of constructs.

Construct	Alpha	CR	AVE
Leaders’ positive mindset	.852	.884	.674
Leaders’ innovative behavior	.948	.950	.829
Covid-19 effect on organizations	.881	.897	.677
Use of ICT	.824	.838	.657
Psychological support	.941	.948	.808

We also assessed the reliability of the scales using principal components analysis (PCA), which was run with a varimax rotation by extracting three factors. The three factors together explained 73.653% of the total variance. As depicted in Table 5, the constructs loaded on the factor that they were theoretically expected to measure with a minimum load of 0.671 (recommended minimum value is 0.60; [62], supporting the unidimensionality of the factors used in this study.

**Table 5:** Results of the principal components analysis.

	Factor 1	Factor 2	Factor 3	Factor 4	Factor 5
PAN-EFF1	−.201	−.040	**.756**	−.218	.017
PAN-EFF2	−.061	.003	**.729**	−.237	−.029
PAN-EFF3	.015	−.075	**.814**	−.015	−.073
PAN-EFF4	−.042	−.035	**.894**	.127	−.122
PAN-EFF5	−.024	−.053	**.873**	−.033	−.074
LEAD-MIN1	.116	.287	−.139	**.671**	.233
LEAD-MIN2	−.003	.405	−.142	**.726**	.147
LEAD-MIN3	.220	.288	.025	**.774**	.052
LEAD-MIN4	.259	.162	−.157	**.785**	.154
INN-BEH1	.292	**.720**	−.088	.276	.337
INN-BEH2	.252	**.765**	−.079	.253	.284
INN-BEH3	.225	**.837**	−.048	.180	.193
INN-BEH4	.229	**.794**	−.067	.353	.222
INN-BEH5	.261	**.821**	.006	.318	.186
ICT-USE1	.016	.092	−.023	.295	**.711**
ICT-USE2	.314	.282	−.099	−.002	**.683**
ICT-USE3	.217	.200	−.072	.133	**.774**
ICT-USE4	.072	.328	−.114	.081	**.810**
PSY-SUP1	**.874**	.070	−.168	.136	.078
PSY-SUP2	**.869**	.268	−.082	.096	.173
PSY-SUP3	**.795**	.289	−.004	.159	.139
PSY-SUP4	**.809**	.341	−.013	.058	.155
PSY-SUP5	**.898**	.107	−.059	.182	.094

Furthermore, we assessed the discriminant validity using the approach described by Bagozzi and colleagues [62], according to which discriminant validity is established when the AVE of each latent construct is higher than the squared correlations of that construct with any other latent construct. As shown in Table 6, all AVE values were superior to the squares of the interconstruct correlations. Therefore, discriminant validity is met for all variables.

**Table 6:** Discriminant validity assessment.

Constructs	Leaders’ positive mindset	Leaders’ innovative behavior	Covid-19 effect on organizations
Leaders’ positive mindset	.674		
Leaders’ innovative behavior	.655	.829	
Covid-19 effect on organizations	−.231	−.169	.677

Note: Values in bold on the diagonal represent the AVE; the other values are the squared interconstruct correlation estimates.

### 4.3. Common method bias

Collecting the data using one survey administered within one single period creates the potential for common method bias [63]. To examine this, we ran Harman’s single factor test in SPSS. We included the three constructs (leaders’ positive mindset, leader’s innovative behavior, and Covid-19 effect on organizations) into a single factor. The results show that the single factor explained 43.93% of the variance, which is less than the maximum threshold of 50%. A common method bias is therefore not a concern in this research.

### 4.4. Hypothesis testing

To test the hypothesized model, a structural model was performed using AMOS 25.0. This model specified leaders’ positive mindset as an independent variable with a direct path to leaders’ innovative behavior. The impact of leaders’ innovative behavior on Covid-19 effect was also tested. Likewise, the use of ICT and psychological support were specified as moderators of the relationship between a leader’s innovative behavior and the effects of the COVID-19 crisis. Along with these hypothesized paths, the structural model included additional paths from the three control variables to the Covid-19 effect on organizations.

**Fig 2:**
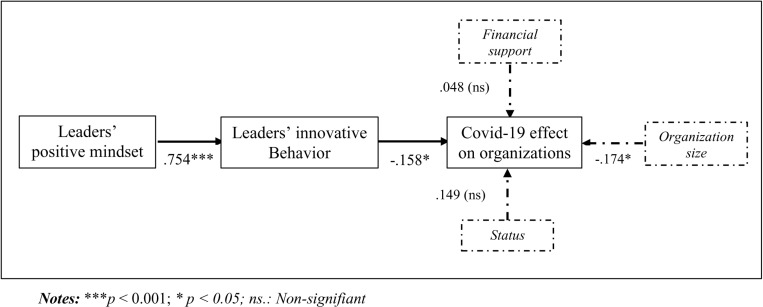
Direct effects results.

**Direct effects:** The SEM analysis reported the statistical indices that follow, indicating a good model fit: χ2/*df* =  1.493; CFI =  0.967; TLI =  0.961; IFI =  0.968; NFI =  0.908; and RMSEA =  0.050. Fig 2 presents the hypothesized model with the standardized path coefficient.

As shown in Fig 2, leaders’ positive mindset has a significant positive impact on a leader’s innovative behavior (β =  0.754, *p* =  .000), supporting H1. Additionally, the results suggest that leaders’ innovative behaviors have a negative relationship with the effects of COVID-19 on their organization, thereby supporting H2 (β =  - 0.158, *p* =  .056).

**Control effects:** As shown in Fig 2, the results regarding the control effects show that only *organization size* has a significant control impact on the effects of the COVID-19 pandemic on organizations (β =  - 0.174, *p* =  .030); the other two control variables (*Public financial support* and *status*) have no significant impact on the Covid-19 effect on organizations (β =  0.048, *p* =  .063 and β =  0.149, *p* =  .547, respectively).

**Moderating effects:** We ran two SEM models to test the moderating effects of using ICT and psychological support. Model A involves assessing the effect of using ICT on the relationship between a leader’s innovative behavior and the Covid-19 effect on their organization. To run the model, we dichotomized our sample using the median of the Likert scale 1–5 responses (4.50), resulting in two groups: group 1 (low use of ICT) with 96 participants and group 2 (high use of ICT) with 69 participants. The estimation of model 1 offered the following indices for the model fit: χ2/*df* =  1.247; CFI =  0.963; TLI =  0.956; IFI =  0.964; and RMSEA =  0.039. All fit indices were above the recommended minimum value, indicating an excellent model fit for the data.

The results of the SEM model support H3, which predicted that the use of ICT moderates the relationship between leaders’ innovative behaviors and the effects of COVID-19 on organizations. As shown in Table 7, this relationship is insignificant for group 1, whose use of ICT was low (β =  -.056, *p* =  .602), and significant for group 2, whose use of ICT was high (β =  - .237, *p* =  .057) supporting H3.

**Table 7:** Results of the moderation effects (*Model A & B*).

Hypothesis	Moderator	Group	Coefficient (β)	p-value
H3	Use of ICT	Low	−.056	.602 (n.s.)
		High	−.237	.057^*^
H4	Psychological support	Low	−.040	.707 (n.s.)
		High	−.283	.038^*^

Notes:

*  p <  0.05; n.s. =  nonsignificant

Model B tests the effect of psychological support to employees on the relationship between leaders’ innovative behaviors and the effects of the COVID-19 crisis on their organizations. We dichotomized our sample into two groups using the median the median of the Likert scale 1–5 responses (4.00), resulting in two groups: group 1 (received low levels of psychological support) with 91 participants and group 2 (received high levels of psychological support) with 74 participants. The estimation of model 1 offered the following indices on the model fit: χ2/*df* =  1.342; CFI =  0.947; TLI =  0.937; IFI =  0.949; and RMSEA =  0.046. All fit indices are above the recommended minimum value, indicating an excellent model fit to the data.

The results of the multi-group analysis support the hypothesized conceptual model predicting that psychological support moderates the relationship between leaders’ innovative behaviors and the effects of COVID-19 on their organization. As displayed in Table 7, this relationship is insignificant for group 1, whose level of psychological support was low (β =  -.040, *p* =  .707), and significant for group 2, whose level of psychological support is high (β =  - .283, *p* =  .038). Thus, hypothesis H4 is supported.

## 5. Discussion and implications

### 5.1. Discussion

We developed and tested a theoretical hypothesized model to examine whether and how a leader’s positive mindset influences leaders’ innovative behavior, and if this behavior, in turn, reduces the effects of the COVID-19 crisis on organizations. Using the situational leadership theory, we proposed that a leader’s positive mindset positively affects leaders’ innovative behavior, which consequently decreases the negative impact of the COVID-19 crisis on organizations. Our findings indeed showed that leaders who exhibited a positive mindset displayed more innovative behaviors, thereby significantly reducing the negative effects of the COVID-19 pandemic on organizations. We also found that both ICT and psychological support to employees played moderating roles in the relationship between leaders’ innovative behavior and the effects of the COVID-19, meaning that a high degree of ICT usage and a high level of psychological support to employees strengthened this relationship.

### 5.2. Implications for theory

This study extends previous research on leadership in several ways. First, our results show that a leader’s positive mindset specifically stimulates a leader’s innovative behavior. Particularly, a mindset that yields positive behavior is more likely to overcome the damage incurred from crises and is a driving force in helping leaders to exhibit innovative behavior, which then helps organizational survival in times of crisis. Several authors have noted that innovation is more likely to be affected by leaders’ behavior. For example, Thayer and al. [64], taking a paradoxical approach to leadership, explained that leaders should create new action strategies and practices to produce innovative solutions, while Zhang and Bartol [65] empirically showed that participative leadership is likely to be effective for fostering leaders’ creativity. Additionally, Prasad and Junni [66] posited that leaders are in an excellent position to promote changes in their organization that support innovative behavior. Given the paucity of leaders’ positive mindset research in the literature, the findings of the various authors presented justify the rationale for our results: that is, leaders who exhibit a positive mindset are more likely to behave innovatively in the context of a crisis.

Second, the current study extends leadership research by examining the impact of leaders’ innovative behavior on the effects that crises, such as the COVID-19 pandemic, have on organizations. Prior research investigated the impact of COVID-19 on leadership behavior [67–69], but our research is among the first to investigate how leaders respond proactively and innovatively during times of crisis (such as during the COVID-19 pandemic) to reduce the negative effects of the crisis on their organization.

Third, the use of ICT moderated the relationship between leaders’ innovative behavior and the effects of the COVID-19 crisis on their organization. Along the same line, Sharma and al. [41] reported that the use of big data analytics and artificial intelligence helped leaders to reduce the negative impact of COVID-19 on their organizations. Moreover, the use of digital tools, designed to cater to the needs of organizations, has helped leaders to promote the exchange of information that serves as an impetus to activate innovative behavior [41,70]. This aligns with our findings that an organization’s use of ICT facilitates the implementation of innovative initiatives, which can lessen the negative effects of the COVID-19 crisis on the organization. However, we add to the literature by showing the moderating role of the use of ICT on the relationship between leaders’ innovative behavior and the effects of the pandemic crisis.

Finally, psychological support to employees moderated the relationship between leaders’ innovative behavior and the effects of the COVID-19 crisis on organizations. Our results show that psychological support to employees is needed in times of crisis to contribute to directing employees’ attention towards making a solid commitment to the implementation of innovative initiatives and help employees overcome the crisis in general. Kniffin et al. [71] contended that the COVID-19 pandemic has created psychosocial barriers in the workplace; they further asserted that leaders must deal with their employees’ demands by providing them with emotional support resources to help them overcome any uncertainties that may hinder their performance in times of crisis. Moreover, Soto et al. [56] stated that providing employees with psychological support allows them to regulate their emotions and respond successfully to crises. Additionally, Barga and Santos [57] put forth that leaders can diminish the harmful effects of a crisis by enhancing employees’ psychological and emotional well-being to help them adapt to the newly emerging settings. These findings provide initial backing for the position that psychological support can strengthen the relationship between leaders’ innovative behavior and the effects of a crisis on their organization.

### 5.3. Implications for practice

Given the scarcity of research on the leaders’ positive mindset in the literature, our study provides important managerial implications for practice. First, we have shown that leaders who display a positive mindset are more likely to enhance their innovative behavior. This result invites leaders to develop a positive mindset as a supportive mechanism associated with innovation. Previous studies proposed that leaders with a positive mindset emphasize the different aspects of the innovation process so that they will be committed to innovation activities, which will help them react rationally in times of crisis [72,73]. Moreover, strengthening the positive mindset of leaders should be an ongoing process in times of crisis, such that a leader might consistently evaluate followers’ psychological well-being through activities like those designed to improve resilience, confidence and stability and enhance adaptability and innovation [30,74]. Hence, as our study supports, leaders seeking innovation during times of crisis should capitalize on their ability to remain positive.

A second managerial implication of our study relates directly to leaders’ innovative behavior in times of crisis. When a crisis, such as the COVID-19 pandemic, arises, leaders must develop their innovation capabilities to handle it and reduce its harmful impacts. In other words, to confront a crisis, leadership matters. Specifically, leaders who exhibit innovative behavior are more likely to assume a key role in crisis management. However, developing and implementing creative activities should not occur as a one-time effort. As Contreras et al. [67] explained, innovative behavior should go along with the different stages of the COVID-19 pandemic, and so innovative behavior should become more routine for leaders throughout the crisis.

Third, our results suggest that leaders’ innovative behavior is a key factor in mitigating the effects of a crisis on organizations when technology is used to a high degree. Research shows that the pandemic required leaders to deeply invest in sophisticated technology, which then had a strong positive impact on their organizations [75,76]. Successful leaders therefore need to effectively use ICT in times of crisis. Hence, leaders should incorporate the most recent advancements in technology to help them curtail the negative impact of crises on them.

Fourth, we found that organization size has a significant and negative control effect on the Covid-19 effect. In other words, organizations with less than 250 employees are more affected by covid-19 than those with more. This is probably because large organizations have more resources to face the pandemic and adapt to the challenges and disruptions that it caused.

Finally, our finding that psychological support moderates the relationship between leaders’ innovative behavior and Covid-19 effect implies that leaders are expected to create a psychological climate that attenuate the negative impact of the pandemic in terms of stress, anxiety, mental health and feelings of insecurity. Gray et al. [77] observed that by prioritizing business practices over emotional demands during a crisis, leaders can trigger more depression, stress and burnout. Instead, leaders should recognize the need to prioritize people’s well-being and avoid any emotional and psychological stressors to improve their organization’s chance of survival in times of crisis [78]. From our findings, we also emphasize that the psychological support of leaders is crucial to facilitating followers’ engagement in implementing innovative initiatives.

## 6. Limitations and future research directions

Our study has several limitations that open avenues for future research.

First, we conducted a cross-sectional study. As a snapshot study, we did not fully capture the lagging effects of leaders’ mindsets, decisions and actions on their organizations during the pandemic. Future research should therefore use longitudinal studies from many countries to better assess how leaders’ positive mindset affects organizational survival and recovery.

Second, our study is based on the Canadian experience of Covid-19 and how leaders’ positive mindset and other factors contributed to their organizations’ survival. In fact, different countries had different responses to the pandemic, including the support provided to businesses to mitigate its effects. As such, our study findings be more applicable to the Canadian context. Future research studies should investigate the same questions in other countries to confirm and potentially expand our model.

Third, our studies may benefit from qualitative methods involving in-depth interviews with leaders on how they mitigated the effects of the pandemic on their organizations. Those qualitative studies would have the advantage of getting into the intricacies and the diversity of leadership actions and decisions to provide rich accounts of organizations’ experience and resilience during the pandemic. On that note, such studies should also investigate what leaders did to get their organizations to recover from the pandemic as quickly as possible.

Fourth, this study was conducted in the context of the Covid-19 pandemic, which was unique in terms of social distancing measures put in place to curb the spread of the virus. There was also massive government support of all kinds to organizations to avoid an economic recession. All organizations were experiencing the effects of the pandemic, even though their responses may vary. However, organizations face other crises that are not pandemic related. Sometimes, an organization may be facing a crisis, for example related to its financial performance, whereas its direct competitors are thriving. Would a leader’s positive mindset make a difference in such a situation? What other leadership ingredients are needed to help an organization recover in that case? Future research should replicate and expand our model to other crises situations.

## Appendix

### Constructs’ measures

**Table:** 

Construct	Item
Leader’s positive mindset	*During the pandemic, the leaders of my organization:* Had a positive mindset, even when facing difficulties Were not easily discouraged by challenges Were confident in the ability of employees. Were confident in the ability of the organization as a whole.
Leaders’ innovative behavior	*During the pandemic, the leaders of my organization:* Developed innovative solutions to our organization’s problems Imagined new ways of operating or doing business Encouraged us to suggest innovative ideas and solutions Strove to find opportunities in the face of challenges Were creative and innovative.
*Covid-19 effect on organizations*	*The pandemic had negatively affected my organization’s:* Internal operations Productivity Market share Financial performance, and Growth.
*Use of information and communication technology (ICT)*	*During the Covid-19 pandemic, my organization:* Effectively used the Internet or other remote work technologies Trained its employees on the effective use of the Internet or other remote work technologies Increased the capacity of its Internet or other remote work technology tools, and Adopted effective practices for using the Internet or other remote work technologies.
*Psychological support*	*During the Covid-19 pandemic, my organization:* Provided psychological support to employees Helped employees deal with stress”, “acknowledged the impact of the pandemic on employees’ mental health Helped employees deal with their personal or family matters Provided support for employees’ mental and physical health.
*Public Financial Support*	*During the pandemic, my organization had financial support from a municipal, provincial, or federal government which was:* Timely Well-targeted Significant Beneficial Practical

## Supporting information

S1 File Dataset_165 responses. (XLS)
